# A Comparative Study of Continuous Versus Interrupted Suturing Technique in Creating a Vascular Access for Hemodialysis: An Institutional-Based Experience

**DOI:** 10.7759/cureus.42004

**Published:** 2023-07-17

**Authors:** Siddhant Roy, Mahakshit Bhat, Nisar Ahmed, Lokesh Sharma, Rajeev Mathur, Vinay Tomar

**Affiliations:** 1 Urology, National Institute of Medical Sciences, Jaipur, IND

**Keywords:** thrill, bruit, patency, dialysis, av fistula

## Abstract

Background

Arteriovenous fistulas (AVFs) are considered the first and best access for patients with end-stage renal disease who need permanent vascular access for hemodialysis over arteriovenous grafts and central venous catheters for reasons that have been well-established. Poor early patency rates pose the biggest challenge in creating vascular access as they cause increased morbidity and economic/psychological concerns among patients. To minimize such effects, it is critical to use a patient-centered approach and carefully choose patients for AVF access creation. This study aimed to compare the primary patency of distal vascular access provided by continuous suturing versus that provided by interrupted suturing.

Methodology

This prospective study was conducted in the urology department of a superspecialty, tertiary care center from November 2021 to November 2022. Patency was assessed immediately after surgery (on the table), one month later, and six months later by palpating thrill and auscultating bruit. A total of 50 patients between the ages of 18 and 70 years who met the inclusion criteria were randomly assigned to two groups of 25 each.

Results

The baseline characteristics of both groups were comparable. At six months (p = 0.09), the continuous suturing group was observed to be somewhat better than the interrupted suturing group, with no significant difference in immediate and one-month patency rates. When compared to the continuous suturing group, the primary patency failure rate was significantly higher in the interrupted suturing group.

Conclusions

Thus, under appropriate circumstances, continuous sutures can be performed with greater ease, resulting in anastomosis that is as patent as that performed with interrupted sutures.

## Introduction

Chronic kidney disease (CKD) and its progression to end-stage renal disease (ESRD) is a major cause of reduced quality of life and premature mortality. Globally, in 2017, there were 697.5 million cases of CKD, with almost one-third of patients with CKD living in two countries, namely, China (132.3 million cases) and India (115.1 cases) [1]. More than one in seven, that is, 15% of US adults or 37 million people, are estimated to have CKD [2]. According to the Kidney Disease: Improving Global Outcomes 2012 clinical practice guideline, CKD is classified into the following five stages considering the glomerular filtration rate (GFR) level [3]: Stage 1: >90 mL/minute GFR denoting kidney damage with normal GFR; Stage 2: 60-89 mL/minute GFR denoting a mild reduction in GFR; Stage 3a: 45-59 mL/minute GFR denoting a moderate reduction in GFR; Stage 3b: 30-44 mL/minute GFR denoting a moderate reduction in GFR; Stage 4: 15-29 mL/minute GFR denoting a severe reduction in GFR; and Stage 5: <15 mL/minute GFR denoting renal failure.

One of the most popular treatment methods for CKD patients is hemodialysis as renal replacement therapy. Arteriovenous fistulas (AVFs), arteri-venous grafts, and central venous catheters (CVCs) are three well-known vascular access methods for hemodialysis. Brescia and Cimino performed the first surgery to create an AVF by subcutaneously anastomosing the radial artery with the neighboring vein [4]. The use of CVC in the internal jugular vein to extend temporary access to hemodialysis began to gain traction in the mid-1980s [5-7], resulting in decreasing AVF utilization and increased use of grafts and CVCs in the 1990s. It resulted in higher patient care expenses, including operations for vascular access failures and complications, as well as increased morbidity and mortality [8-10]. To increase the use of AVF in the United States, CMS and ESRD launched the Fistula First Initiative [11]. The initial goal was to increase the appropriate use of AVF for hemodialysis access and meet or exceed the Kidney Disease Outcomes Quality Initiative (KDOQI) guidelines of 50% AVF use in incident patients (first placed access) and 40% AVF use in prevalent patients (previous surgically created access) [8]. Because the 40% AVF usage objective in prevalent patients was met ahead of schedule, CMS raised the quality goal for AVF use in prevalent patients to 65% [11]. KDOQI believes that when estimated GFR (eGFR) is 15-20 mL/minute/1.73 m^2^ in no dialysis CKD patients with a gradual decline in renal function, referral for dialysis access assessment and subsequent creation should occur. Patients with unstable and/or rapid eGFR decline (>10 mL/minute/year) should be referred earlier [12]. CVC use in CKD patients has substantial implications, including a higher risk of infection [13-15], hospitalization [16], CVC dysfunction [17], central venous stenosis [18,19], and mortality [20,21]. One of the reasons that can explain no improvement in CVC rates is the fact as more vascular access is created, it starts serving as bridge access until the vascular access is ready for hemodialysis.

If the distal vascular access fails or the wrist has weak vasculature, the standard practice recommendation is to start distally by constructing radiocephalic fistulas and then proceeding proximally to provide elbow brachiocephalic, brachiobasilic vascular access [22]. One of the most worrying aspects of vascular access creation is the low rate of early patency [23]. Primary patency rates range from 50% to 80% [24]. Failures in vascular access result in CKD patients being hospitalized, increasing morbidity and death. Failures of vascular access have both economic and psychological consequences for patients. It subjects patients to denial of functional access and a reduction in the number of sites, as well as further treatments to salvage AVF failures. To avoid such consequences, it is important to follow a more patient-centric approach and select patients appropriately for AVF access placement.

AVF maturation failure and delayed maturation are caused by various factors, including inadequate arterial and venous diameter, calcified arteries, intimal hyperplasia, stenosis, improper anastomosis, and patient hemodynamics [25]. Various operational alterations, such as side-to-side anastomosis and adjustments in anastomotic angles to eliminate venous kinks, have been proposed to avoid AVF failure and enhance flow. With no reduction in CVC usage in a patient, Fistula First Breakthrough Initiative (FFBI) shifted its focus to CVC reduction and launched the “Fistula First - Catheter Last Initiative” [26]. FFBI failed to assess patients for whom the risk of AVF development outweighed the risk of not using a patient-centric approach. Recent data have challenged these associations because of the high complication rates of AVF maturation failure requiring interventions and have prompted a re-evaluation of the Fistula First approach [11].

The 2019 KDOQI vascular access recommendations recommend the ESKD Life Plan, which takes a more patient-centric approach to hemodialysis vascular access and takes into account numerous aspects of a patient’s needs and dialysis access eligibility. Furthermore, the KDOQI Vascular Access Committee stressed that the overall goal for a hemodialysis patient should be a functioning AVF, rather than placing AVF in patients with a low possibility of maturation or usability.

A more patient-centered approach will necessitate a greater emphasis on the subset of patients who are unlikely to benefit from AVF placement, such as elderly ESRD patients, patients with poor vasculature (e.g., calcification and small arteries and veins), patients with slowly progressive CKD who are more likely to die than progress to ESRD, and patients with poor overall health and prognosis and limited life expectancy. Furthermore, the patient’s quality of life, comfort, and contentment should be prioritized over the aims and clinical outcomes of AVF.

Our study aimed to compare the primary patency (assessed by the presence of thrill and bruit) of the distal vascular access created by the continuous suturing technique to that created by the interrupted suturing technique using various suturing techniques, each with its own set of advantages and disadvantages.

## Materials and methods

This prospective observational study was conducted in the urology department of a superspecialty, tertiary care center from November 2021 to November 2022. During this period, a total of 50 patients were included in the study. Patients between the ages of 18 and 70 who were scheduled for primary radiocephalic AVF surgery were evaluated and enrolled in the study. Patients who did not provide consent, those who had a history of previous AVF surgery, those with AVFs formed as a result of the failure of primary AVF during the study period, and those above the age of 70 years were excluded. A history of diabetes, hypertension, coronary artery disease, tuberculosis, anticoagulant therapy, previous surgical intervention, intravenous cannulation, and needle prick within the past 14 days on the operative forearm was noted. A history of dialysis and previous dialysis access was also noted. Non-dominant hand of the patient was given preference. Local examination of the operative forearm included the Allen test, incision scar marks for previous fistula surgery, the presence of edema, previous cannulation, needle prick site, signs of thrombophlebitis, and cellulitis. A preoperative color Doppler examination was performed to determine the diameter of the cephalic vein and radial artery. In all cases, an end-to-side anastomosis was performed. Medical records were used to store preoperative, operative, and postoperative data. After receiving informed written consent, patients were randomly assigned to one of two groups using the lottery method. Group 1 was subjected to continuous suturing, while group 2 was subjected to interrupted suturing. The primary endpoint, which was determined by palpating thrill and auscultating bruit at six months, was used to examine the result. Patency at one month and immediately postoperatively (on the table) were secondary goals.

Operative technique

A 6-7 cm transverse S-shaped incision was made at the junction of the proximal and distal two-thirds of the non-dominant forearm. The cephalic vein and radial artery were appropriately mobilized and skeletonized. An arteriotomy of approximately 6-7 mm in size was performed, and the end of the cephalic vein was anastomosed with the side of the radial artery using 8-0 polypropylene sutures, followed by a non-compressive dressing. During the postoperative period, each patient was given an Ecosprin 75 mg tablet once daily for 14 days. Counseling was provided, and all patients were instructed to begin hand-ball exercises on postoperative day one. The administration of a blood pressure (BP) cuff and measurement, intravenous cannulation for medicine, and blood collection in the operative hand, as well as pressure avoidance, were explained to each patient. For six months, all patients were seen in the outpatient department. Nephrologists determined fistula usability; typically, hemodialysis commences after four weeks of fistula formation in our setting, provided vascular access is functional, as determined by palpating thrill.

Statistical analysis

SPSS version 22 (IBM Corp., Armonk, NY, USA) was used for data analysis. All continuous variables were presented as mean and standard deviation, while categorical data were presented as frequency and percentage. The t-test was used to evaluate continuous data, and the chi-square test was used to investigate categorical data. A p-value of 0.05 was considered significant.

Ethical approval

The study conformed to the ethical norms and standards of the Declaration of Helsinki, including the local ethics committee approval. Study approval was obtained from the Institutional Ethics Committee, National Institute of Medical Science and Research (approval number: ECR/665/Inst/RJ/2014/RR-17/2022).

## Results

A total of 50 patients were enrolled in the study and were divided into two cohorts of continuous and interrupted suturing techniques, with each cohort comprising 25 patients. The mean age of patients in the interrupted group was 47.88 years while that in the continuous group was 44.8 years (p = 0.437). Overall, 80% (n = 40) of the patients were males and 20% (n = 10) were females. The groups were comparable in age and sex in both cohorts. Most patients had hypertension as the cause of renal failure in both cohorts (p = 0.002), with diabetes mellitus being the second most common cause (p = 0.03), and ischemic cardiomyopathy, dilated cardiomyopathy, Autosomal dominant polycystic kidney disease, and dengue among other causes (Table 1).

**Table 1:** Baseline patient characteristics with results presented as a percentage of the total population, n (%). P-values <0.05 are significant. CAD: coronary artery disease; DCMP: dilated cardiomyopathy; DM: diabetes mellitus; HTN: hypertension

	Continuous	Interrupted	P-value
Mean age (years)	44.8	47.88	0.437
Sex			
Female	3 (12)	7 (28)	0.157
Male	22 (88)	18 (72)	
Comorbidities			
CAD	0 (0)	1 (4)	0.48
DCMP	0 (0)	1 (4)	0.48
DM	8 (32)	4 (16)	0.03
HTN	15 (60)	17 (68)	0.002
Ischemic cardiomyopathy	0 (0)	1 (4)	0.48
Seizures	1 (4)	(0) 0	0.48
None	1 (4)	1 (4)	0.47

The mean preoperative arterial diameter was marginally higher in the continuous arm at 2.67 mm compared to the interrupted cohort at 2.43 mm (p = 0.02), whereas the mean cephalic vein diameter in the continuous cohort was 2.44 mm compared to the interrupted arm at 2.52 mm (p = 0.65). On-table thrill on palpation was 84% in the continuous versus 92% in the interrupted arm (p = 0.38), whereas on-table bruit on auscultation was 88% in the continuous versus 96% in the interrupted arm (p = 0.297) (Table 2).

**Table 2:** Preoperative radial artery and cephalic vein diameter with data presented as mean (standard deviation). P-values <0.05 are significant.

	Continuous	Interrupted	P-value
Radial artery diameter (mm)	2.67 (0.41)	2.43 (0.32)	0.0262
Cephalic vein diameter (mm)	2.44 (0.71)	2.52 (0.52)	0.6515
On-table thrill	84 (21)	92 (23)	0.384
On-table bruit	88 (22)	96 (24)	0.297

Primary patency at six months was marginally higher in the continuous arm (76% in the continuous arm versus 72% in the interrupted arm; p = 0.097). However, immediate patency (96% versus 88%; p = 0.33) and patency at one month (92% versus 80%; p = 0.28) were marginally higher in the interrupted group compared to the continuous group (Table 3).

**Table 3:** Comparison of patency rates with results presented as a percentage of the total population, n (%). P-values <0.05 are significant.

	Continuous	Interrupted	P-value
Immediate patency	22 (88)	24 (96)	0.333
Patency at one month	20 (80)	23 (92)	0.284
Patency at six months	19 (76)	18 (72)	0.097

## Discussion

**Figure 1:**
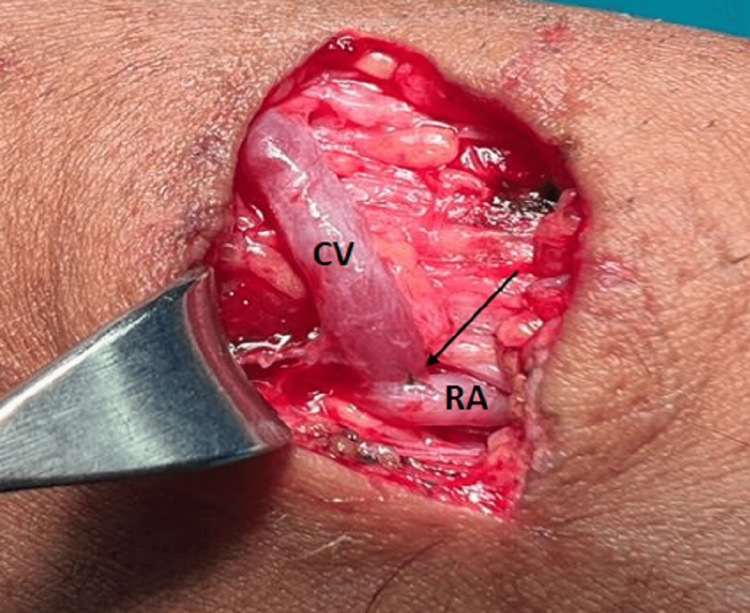
End-to-side arteriovenous fistula anastomosis with continuous suturing. Note the anastomotic site (black arrow). CV: cephalic vein; RA: radial artery

**Figure 2:**
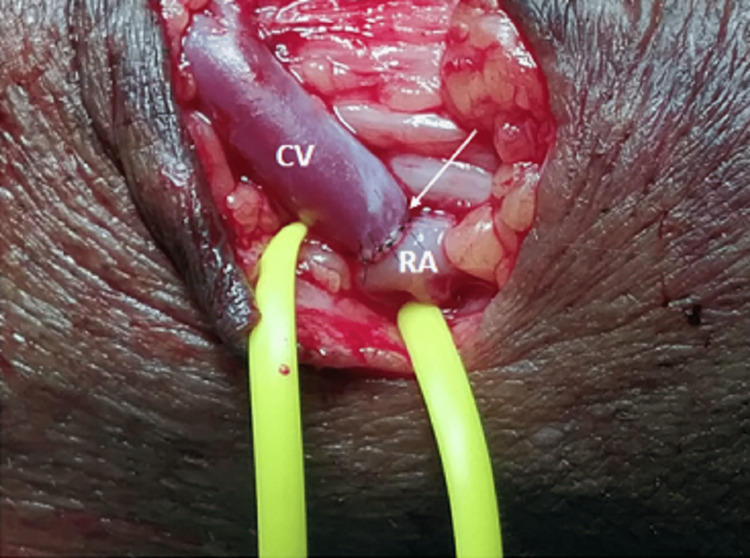
End-to-side arteriovenous fistula with interrupted suturing. Note the anastomotic site (white arrow). CV: cephalic vein; RA: radial artery

For the development of radiocephalic fistulas, our data show no significant difference between the continuous suturing technique and the interrupted suturing technique. In our study, the continuous suturing approach demonstrated somewhat better primary patency at six months (76% versus 72%; p = 0.09) than the interrupted group. Laskar et al. [27] reported similar results in their investigation. However, the interrupted group had higher immediate patency (96% versus 88%; p = 0.33) and one-month patency (92% versus 80%; p = 0.28). Although interrupted sutures are time-consuming, there is a theoretical risk that interrupted sutures bleed more from the gaps between the suture lines, whereas continuous sutures, while less time-consuming, carry the risk of tightening and puckering along the suture lines, making continuous sutures unsuitable for radiocephalic fistulas with vessels smaller than 2 mm [28,29]. On-table excitement and bruit are long-term indices of primary patency [30], and comparable findings were observed in this study. The life expectancy of CKD patients has increased as awareness of the disease has grown along with research in the field of hemodialysis. As a result, such patients would require multiple vascular accesses throughout their lives. Unless the wrist vasculature is weak, it is always prudent to save as many vessels as feasible and to begin with distal vascular access [22]. Permanent vascular access is critical in the management of these patients. The goal should be to create a functional and well-functioning AVF that can provide enough dialysis with minimal problems (Figures 1, 2). The main AVF failure rates range from 20% to 60% [30], which is comparable to the current study, which had a main failure rate of 26% in both cohorts combined.

**Figure 3:**
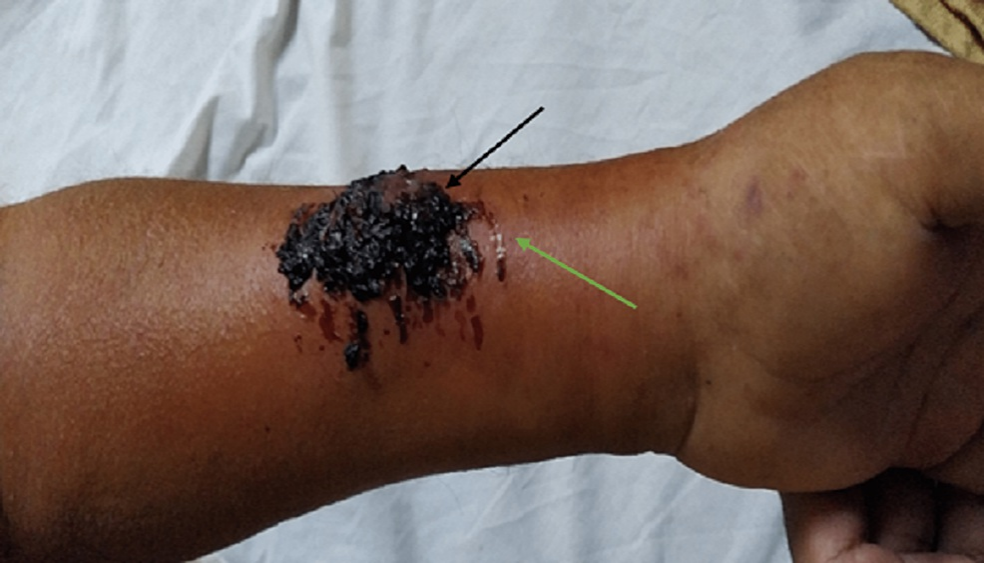
Arteriovenous fistula failure due to trauma. Note blood clots (black arrow) with the surrounding area showing cellulitis (green arrow).

**Figure 4:**
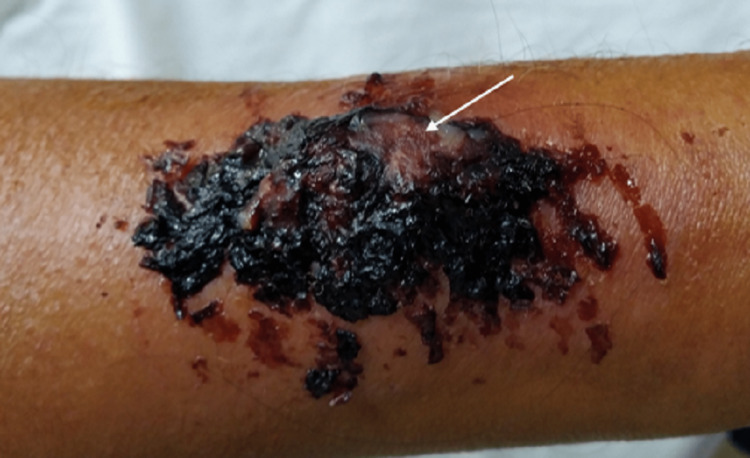
Arteriovenous fistula failure due to trauma. Note the area of impending rupture (white arrow).

In both the cohorts combined, four patients (three in the continuous arm and one in the interrupted arm) were re-explored immediately due to the absence of thrill and bruit (on-table failure). These patients underwent brachiocephalic fistula surgery on the ipsilateral arm in the same setting. The above revised proximal fistulas were excluded from the study. By the one-month follow-up, three patients (two in the continuous arm and one in the interrupted arm) had non-functioning AVF. All these patients underwent brachiocephalic fistula on the ipsilateral arm. One patient in the interrupted arm had an early failure due to trauma and developed a hematoma with impending rupture, which led to re-exploration, clot evacuation, and ligation of the bleeding vessel in an emergency (Figures 3, 4).

After six months, six patients (one in the continuous arm and five in the interrupted arm) failed. Four underwent brachiocephalic fistula surgery and two underwent radiocephalic fistula surgery proximal to the site of earlier surgery on the ipsilateral arm. Early failure (fistulas that never develop or fail within three months) in both arms was probable due to calcified arteries, venous outflow stenosis, trauma, and the inability to follow postoperative instructions such as handball exercises, and non-compliance with anticoagulant therapy. The cause of late failures (fistulas that fail after three months of successful use) in three individuals was unknown; however, it was likely post-dialysis hypotension that caused the fistulas to fail.

This study has some limitations such as the small sample size and the single-institution design of the study.

## Conclusions

We hypothesize that the presence of on-table thrill and bruit is a strong predictor of effective AVF. Preoperative clinical and radiological assessment of vasculature of candidates for AVF formation is critical. Based on our observations, there is no discernible difference between the continuous and interrupted suturing techniques. Although the continuous suturing technique cohort performed marginally better than the interrupted arm in our study, the main patency rate in the interrupted cohort declined dramatically when compared to the continuous arm. Thus, under suitable conditions, continuous sutures can be performed with more ease and anastomosis that is as patent as those conducted with interrupted sutures. Postoperative guidelines such as handball exercises, anticoagulation therapy, avoiding pressure on the operated arm such as BP measurement, avoiding intravenous cannulation, and refraining from arm vein sampling from the operated arm must be rigorously followed.
